# Influences of Efficient Spraying of Cement-Based Slurries on Recycled Coarse Aggregate

**DOI:** 10.3390/ma15217730

**Published:** 2022-11-02

**Authors:** Jinming Yin, Aihong Kang, Peng Xiao, Zhengguang Wu, Changjiang Kou, Yongfan Gong, Chenghui Xiao

**Affiliations:** 1 College of Civil Science and Engineering, Yangzhou University, Yangzhou 225100, China; dx120200085@yzu.edu.cn (J.Y.); xpyzu@163.com (P.X.); zgwu@yzu.edu.cn (Z.W.); changjiang.kou@yzu.edu.cn (C.K.); yfgong@yzu.edu.cn (Y.G.); 2 Taizhou Institute of Science and Technology, Nanjing University of Science and Technology, Taizhou 225300, China; 3 Yangzhou Huimin Renewable Resources Co., Ltd., Yangzhou 225100, China; 15996009709@126.com

**Keywords:** recycled coarse aggregate, enhancement, spraying method, immersing method, crushing value, water absorption, apparent density

## Abstract

The inferior property is usually one of the major problems of recycled coarse aggregate (RCA), and the utilization of the RCA is limited. Strengthening the RCA is being widely explored. Immersing the RCA in the cement-based slurry is an effective approach. However, lots of slurry and time are required, and it is difficult to integrate the immersing method into the production line of the RCA. In this paper, a circular spraying method was proposed to treat the RCA using cement-based slurry. The immersing method was also conducted to verify the feasibility of the spraying method. The crushing value (CV), 24 h water absorption (WA), apparent density (AD) and dynamic water absorption (DWA) were tested, and the micro-morphology was also observed to explore the strengthening mechanism. Results showed that the CV and the WA decreased by up to 30.0% and 14.3% when the spraying method was used. The AD was slightly influenced by the cement-based slurry regardless of the treatment method. Considering the CV, WA and AD, the comprehensive grade of the RCA could be enhanced from III to II by using the spraying method. It was worth noting that the effects of the spraying method and the immersing method were basically equivalent. When the spraying method was adopted, only about 1 min and a small amount of slurry (about 5% of the RCA mass) were required to treat the RCA.

## 1. Introduction

An increasing amount of construction and demolition waste (CDW) is being generated all around the world, especially in developing countries. At the same time, the consumption of natural aggregates crushed from stones is increasing rapidly. In order to address the environmental and resource issues, many researchers have been devoted to research on the recycling of CDW [1,2,3,4,5,6,7,8].

Producing recycled coarse aggregate (RCA) is a promising way to reuse CDW. Due to the adhered mortar, RCA, produced with concrete waste, has been labeled as having high porosity, high crushing value and high water absorption. Therefore, recycled aggregate concrete (RAC), incorporated with RCA, is inferior to normal aggregate concrete (NAC) in many aspects, such as compressive strength, chloride resistance and water absorption [9,10,11,12,13,14].

In order to improve the properties of RCA, various treatment methods have been attempted. At present, there are mainly three approaches: (a) eliminate the old mortar by grinding, heating grinding or acid solution et al. [15,16,17,18,19]; (b) strengthen the old mortar with CO_2_, sodium silicate or nano materials et al. [1,20,21,22,23,24,25]; (c) coat the surface by immersing RCA in cement-based slurries such as cement, fly ash and silica fume et al. [26,27,28,29,30]. All the above approaches can improve the properties of RCA to a certain extent. However, there are also some problems that need to be focused on, such as environmental risk and energy consumption [31,32]. Based on this, immersing RCA into the cement-based slurry is a promising treatment method because it does not need extra energy and the environmental risk is low; furthermore, the materials are easily available, and the cost is low. Martirena F et al. [27] used Portland cement to prepare slurry with water to cement ratio (W/C) of around 0.5. Four to eight millimeter RCA was adopted and coated for 5 min. A total amount of 163 kg cement was needed for 1 m^3^ RCAs. They found that about 0.2 mm cement layer was formed on the treated RCA, freeze-thaw resistance was enhanced and 24 h water absorption (WA) was decreased up to 55%. Fubo CAO et al. [28] used the slurry containing cement, rice husk ash and metakaolin to treat RCA. RCA was immersed in slurry for 1 h, and the results showed that the crushing value and the porosity decreased by up to 1.47% and 3.4%, respectively; however, the WA increased. When the treated RCA was used in RAC, the strengths of RAC were all enhanced. Cement and nano-SiO_2_ were adopted by Zhang Hu et al. [33] to prepare a slurry in which RCA was immersed for 45 min. They found that the apparent density was not influenced obviously, but the crushing value (CV) and the WA were all reduced. Shaban W M et al. [31] used cement, fly ash and nano silica fume to prepare cement-based slurries, of which the binder to water ratio (b/w) was 0.1 to 0.3. Then, RCA was immersed for 1 h to 4 h. Superior effects on the RCA were shown when cement was incorporated in the slurries.

Although the positive effects of cement-based slurry on the RCA have been proved by many researchers, there are still some disputes. On the other hand, immersing method would consume too much slurry (twice the weight of the RCA [31]) with little slurry absorbed, and the immersing (coating) time is up to 4 h. Therefore, it is difficult to apply the immersing method to engineering practice. Recently, a spraying treatment method has been reported, but the RCA was still treated in a container; just like the immersion method, more slurry was needed, and the influence of the spraying method was not deeply assessed [12,20].

According to the Chinese code of *Specification for Mix Proportion Design of Recycled Concrete* (DB37/T 5176-2021), the higher the grade of the RCA is, the wider its application is. Hence, this study aimed to improve the properties of the RCA by using a novel spraying method, which could save a lot of material and time. Slurry absorption of the RCA was tested to determine an optimum spraying procedure. The properties, including slurry absorption, apparent density (AD), CV, WA and dynamic water absorption (DWA), of the RCA treated by immersing method and the spraying method were assessed and compared. Furthermore, micro morphology was observed to explore the influencing mechanism. The effective spraying method, which is easier to integrate with the RCA production line and consumes less material and time, may provide an alternative to enhance the RCA.

## 2. Materials and Methodology

### 2.1. RCA

**Figure 1:**
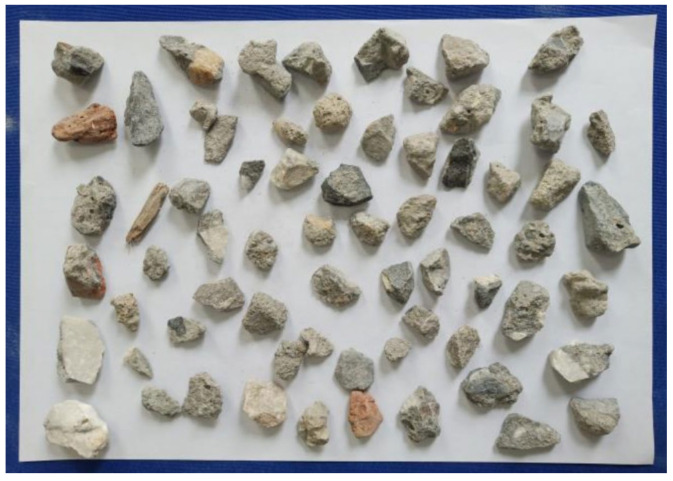
The raw RCA.

**Figure 2:**
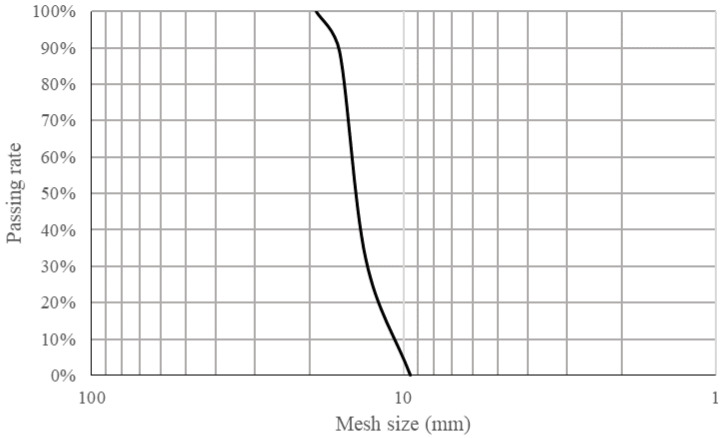
The grading curve of the RCA.

In this study, the 9.5 mm–19 mm RCA was used, which was provided by Yangzhou Huimin Renewable Resources Co., Ltd. in Yangzhou, China. The information and property of the raw concrete were unknown. There were few crushed brick aggregates in the RCA, as shown in Figure 1, and the grading curve is shown in Figure 2. The RCA was washed and air-dried before treatment and testing. By using the high-temperature calcination method, the mortar adhesive rate was about 31.2%. The main properties of the raw RCA are shown in Table 1. According to the Chinese code of *Recycled coarse aggregate for concrete* (GB/T 25177-2010), the grade of the RCA is also listed in Table 1. It showed that the comprehensive grade of the RCA was III, which meant the utilization would be strictly limited.

### 2.2. Cement

An ordinary Portland cement (42.5) was adopted, which was produced by Taizhou Yangwan Hailuo Cement Co., Ltd. The cement met the Chinese standard of *Common Portland Cement* (GB175-2007), and the main chemical compositions are shown in Table 2.

### 2.3. Silica Fume

Silica fume (SF) was supplied by Zhengzhou Hengnuo Filter Material Co., Ltd., Zhengzhou, China. The average particle size was 0.1–0.3 μm, and the chemical compositions are shown in Table 3.

### 2.4. RCA Treatment Methods

In this study, an immersing treatment method and a circular spraying treatment method with cement-based slurry were all conducted. The proportions of cement-based slurries used in this study are shown in Table 4. In order to assess the effect of the spraying method, the raw RCA (denoted as RCA0), I_C1.0 and I_CS1.0 were controls.

By using the immersing treatment method, enough cement-based slurry (more than twice the mass of the RCA) was prepared to ensure the RCA could be fully immersed, and the immersing time was set to 40 min. Then, the RCA was taken out and cured with RH larger than 90% at room temperature for 28 days. Finally, the RCA was air-dried for 2 days before testing.

**Figure 3:**
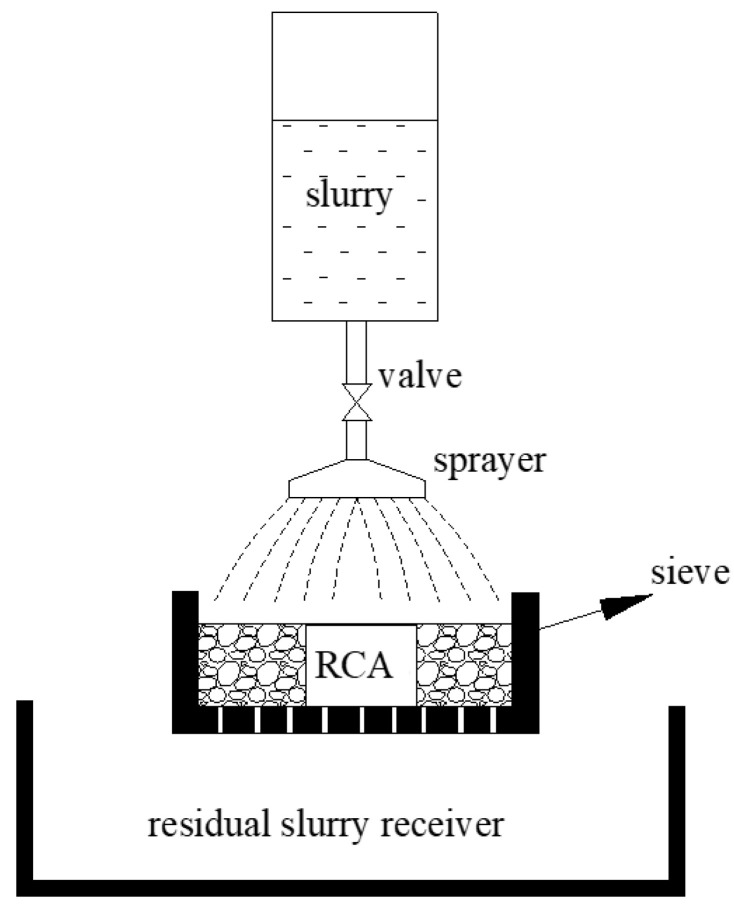
Schematic of the spraying equipment.

**Figure 4:**
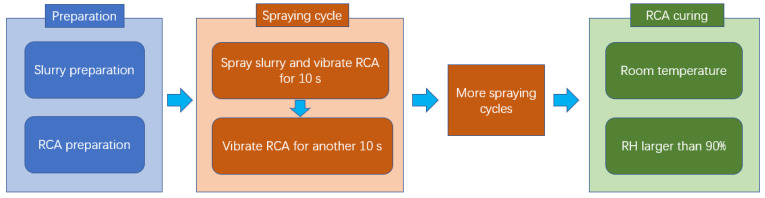
The procedure of the circular spraying treatment method.

**Figure 5:**
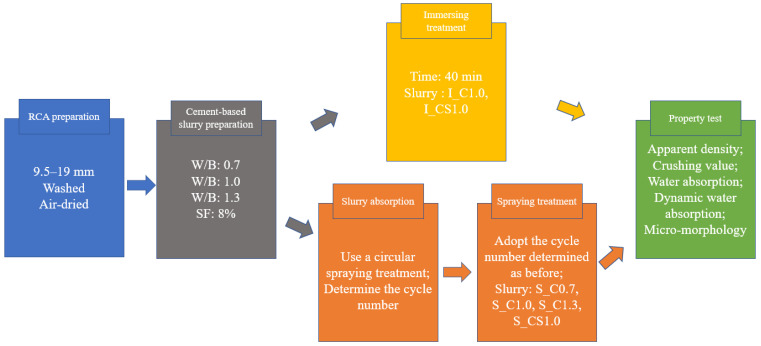
The procedure of the experiment.

Regarding the circular spraying treatment, the cement-based slurry was prepared and filled in spraying equipment, as shown in Figure 3. Then, a batch RCA of around 1.5 kg was put into the sieve, and the spraying flow rate was 5 g/s per kilogram RCA. The circular spraying treatment method contained one or more spraying treatment cycles, and one spraying cycle was conducted as follows: (a) spray cement-based slurry on the RCA for 10 s and vibrate the RCA at the same time; (b) stop spraying slurry and vibrate the RCA for another 10 s to make the RCA evenly coated. The number of the spraying treatment cycle would be determined by the slurry absorption result. After the spraying treatment, the RCA curing method was the same as the immersing treatment. The procedure of the circular spraying treatment method is shown in Figure 4, and the procedure of the experiment is shown in Figure 5.

### 2.5. CV Test

According to the Chinese national standard *Pebble and crushed stone for building* (GB/T 14685-2011), the CV of the RCA was tested. The load rate was 1 kN/s, and the maximum load was 200 kN. The CV was calculated by the following equation:

(1) $$Q_{c}=\frac{G_{1}-G_{2}}{G_{1}}\times 100$$

where *Q_c_*, CV, is the crushing value, %; *G*_1_ is the mass of the RCA before loading, g; *G*_2_ is the mass of the RCA remaining on the 2.36 mm mesh after loading, g.

### 2.6. WA Test and DWA Test

In order to obtain comprehensive insight into the property of the water absorption of the RCA, the WA and the DWA were tested in this study. The WA was tested according to the Chinese national standard *Lightweight aggregates and its test methods—Part 2: Test methods for lightweight aggregates* (GB/T 17431.2-2010). The WA was calculated by the following equation:

(2) $$w_{a}=\frac{m_{0}-m_{1}}{m_{1}}\times 100$$

where *w_a_*, WA, is the 24-h water absorption, %; *m*_0_ is the mass of the RCA immersed in water for 24 h, g; *m*_1_ is the mass of the RCA dried by an oven, g.

Following the references [23,34], the DWA test was performed. Hydrostatic balance was used, and the water absorption at time *t* was calculated using Equations (3) and (4):

(3) $$w_{at}=\frac{m_{t}-m_{s}}{m_{1}}\times 100$$

(4) $$m_{s}=m_{24}-w_{a}\times m_{1}$$

where *w_at_* is the water absorption of the RCA at time *t*, %; *m_t_* is the mass of the RCA immersed in water at time *t*, g; *m_s_* and *m*_24_ are the masses of the RCA immersed in water at time *t* = 0 and *t* = 24 h, g, respectively.

### 2.7. AD Test

The AD of the RCA was tested in accordance with the Chinese national standard *Pebble and crushed stone for building* (GB/T 14685-2011). The AD was determined with Equation (5):

(5) $$\rho_{0}=\left(\frac{G_{0}}{G_{0}+G_{2}-G_{1}}\right)\times \rho_{w}$$

where *ρ*_0_, AD, is the apparent density of the RCA, kg/m^3^; *G*_0_ is the mass of the RCA dried by an oven, g; *G*_1_ is the mass of the RCA and basket hanged in water, g; *G*_2_ is the mass of basket hanged in water, g; *ρ_w_* is the density of water, kg/m^3^.

### 2.8. Micro-Morphology Analysis

A field emission scanning electron microscope (GeminiSEM 300), manufactured by Carl Zeiss Microscopy GmbH (Jena, Germany), was used to observe the micro-morphology of the RCA. The RCA was sputter-coated with gold-palladium prior to imaging. The acceleration voltage was set to be 10 kV. In order to obtain a universal conclusion, more than 6 images were taken for each RCA, and the representative images were shown in this paper.

## 3. Results and Discussion

### 3.1. Slurry Absorption

**Figure 6:**
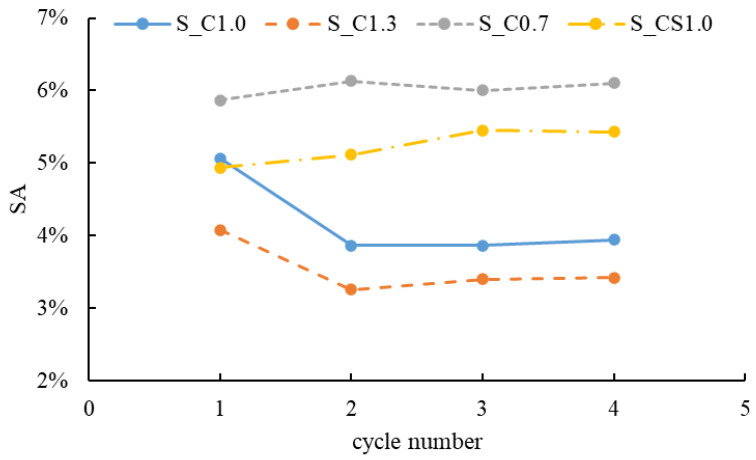
Relationships between the SA and the spraying cycle number of different slurries.

**Figure 7:**
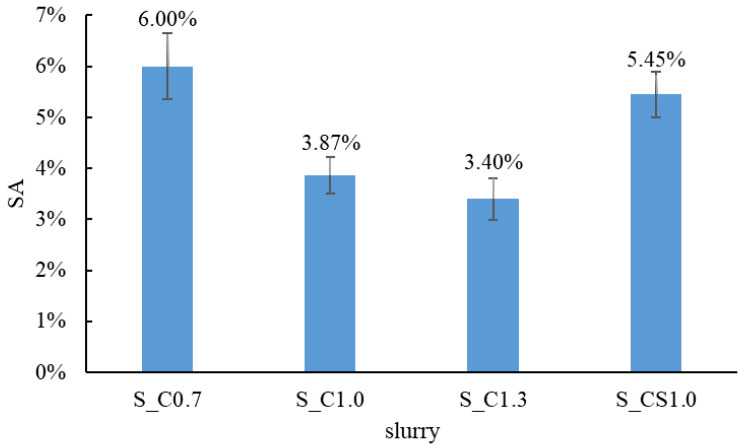
The SA of different slurries with 3 spraying cycles.

When the RCA was treated with the spraying method, the mass of the slurry absorbed by the RCA during each treatment cycle was recorded, and the time-varying slurry absorption (SA) could be analyzed. The SA results of different slurries are shown in Figure 6. For S_C1.0 and S_C1.3, the SA showed a decreasing trend with the increase in the cycle number. In contrast, the SA of S_C0.7 and S_CS1.0 increased with the increase in the cycle number. In general, the order of the SA from large to small was S_C0.7, S_CS1.0, S_C1.0 and S_C1.3. The reason was that when W/B was higher, there was more water in the slurry, and the water was quickly absorbed by the RCA during the first spraying cycle. Therefore, cement particles remained on the surface of the RCA, leading to a higher concentration of the slurry. With the increase in the spraying cycle, when the lower concentration slurry was sprayed onto the surface of the RCA, less water could be absorbed by the RCA and the higher concentration slurry was diluted, and some cement particles were washed away. With the W/B decreasing, the slurry became denser and more slurry was absorbed; regarding S_CS1.0, 8% SF was incorporated, and the slurry was denser than pure cement slurry. Hence, the denser the slurry was, the more the slurry was absorbed. When the cycle number reached 3, the SA tended to be steady for all slurries. It meant that the SA did not always increase with the increase in the spraying treatment time. In this study, 1 min (3 spraying cycles) was enough, and more spraying treatment time meant waste, not only time but also material. The SA of different slurries with three spraying cycles is shown in Figure 7. From this aspect, the spraying method was more efficient than the immersing treatment, and three spraying cycles were adopted in the next tests.

**Figure 8:**
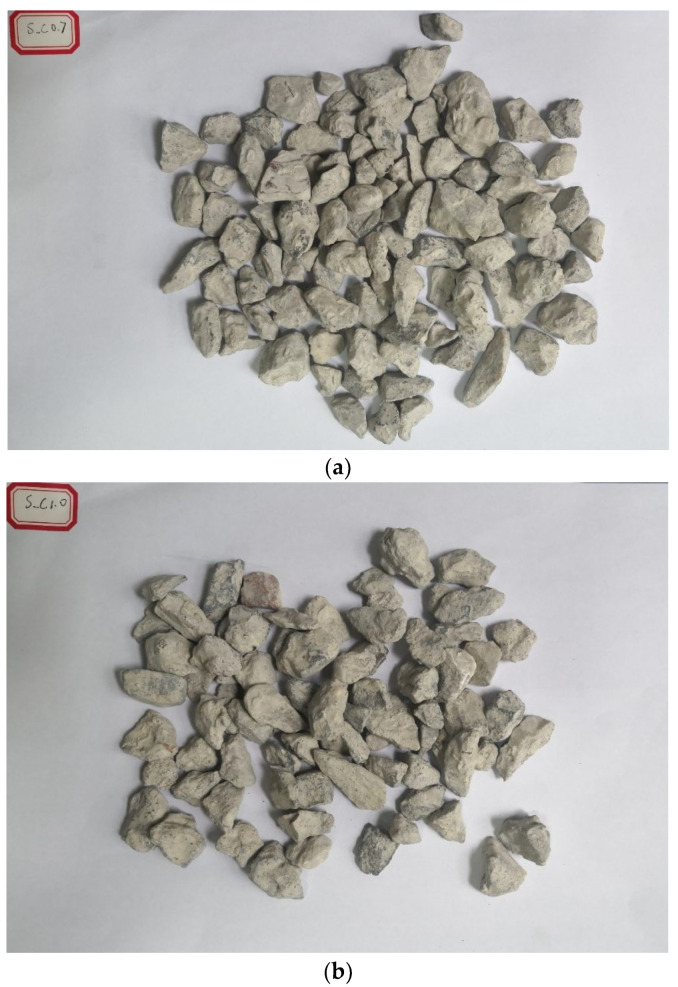

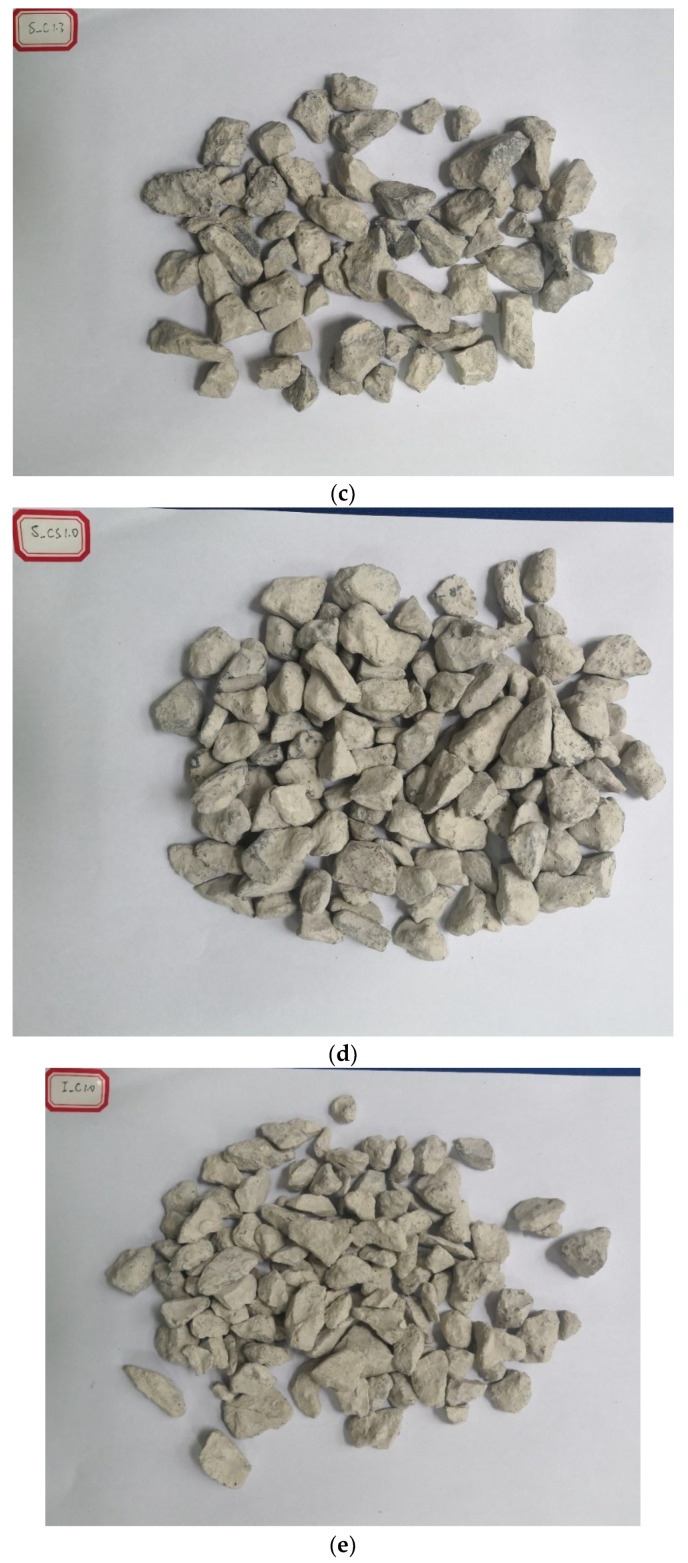

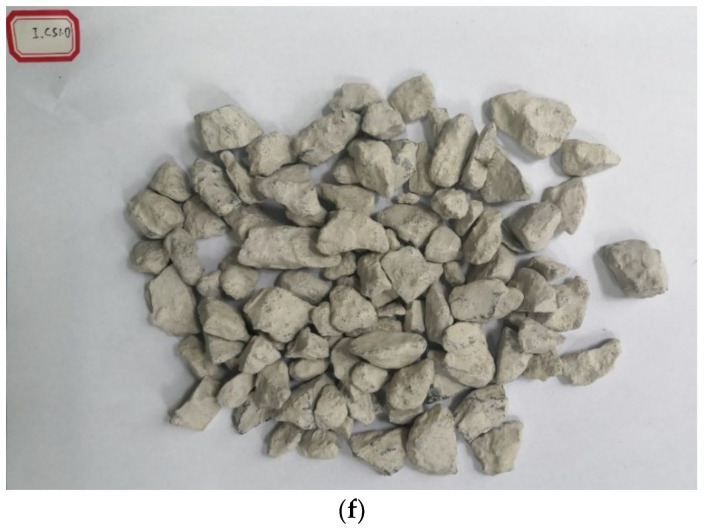
Images of the treated RCA. (**a**) S_C0.7; (**b**) S_C1.0; (**c**) S_C1.3; (**d**) S_CS1.0; (**e**) I_C1.0; (**f**) I_CS1.0.s.

All the treated RCA are shown in Figure 8. It could be found that a hardened coating layer was formed for all the treated RCA and the surface became smoother than the raw RCA. The larger the W/B was, the thicker the hardened cement layer was. When 8% SF was incorporated, the coating layer was even thicker. By comparing S_C1.0 with I_C1.0 and S_CS1.0 with I_CS1.0, the coating effect was basically the same, which meant that there was no obvious difference between the spraying method and the immersing method.

### 3.2. AD

**Figure 9:**
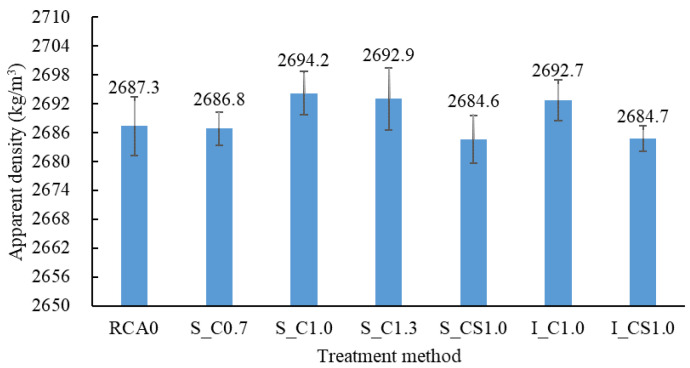
Apparent density of the RCA.

**Figure 10:**
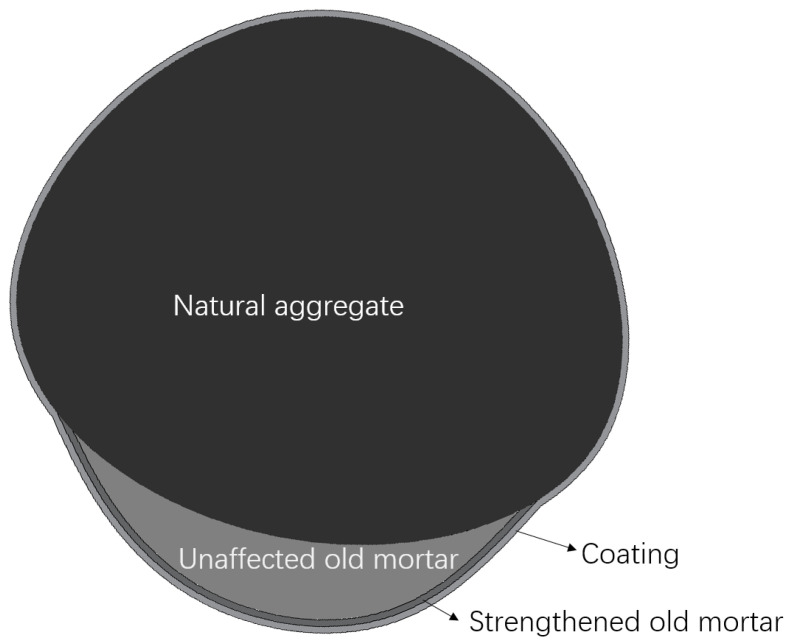
Schematic of the treated RCA.

The AD results of the treated RCA are shown in Figure 9. It could be found that the AD of S_C1.0, S_C1.3 and I_C1.0 was higher than that of the raw RCA, while the density of S_C0.7, S_CS1.0 and I_CS1.0 was lower than that of the raw RCA. It indicated that the density decreased with the decrease in the W/B and the incorporation of SF. The reason was that there was natural aggregate, unaffected old mortar, strengthened old mortar and coating within the treated RCA, as shown in Figure 10. The strengthened old mortar resulted in higher density, while the coating resulted in lower density [35,36]. When the W/B decreased or the SF was incorporated, the more cement-based slurry was absorbed by the RCA, which led to a thicker coating; therefore, the density was decreased. However, according to the classification of the RCA, the grade of all the treated RCA was I (higher than 2450 kg/m^3^). By comparing the result with others [28,37], the effect of cement-based slurry on the density of the RCA was related to the density of the raw RCA. When the density of the raw RCA was lower, as shown in [28,37], the density of the treated RCA tended to be higher

When comparing S_C1.0 with I_C1.0 and S_CS1.0 with I_CS1.0, the difference in the AD between the spraying method and the immersing method was not obvious. It indicated that, in terms of AD, the effect of the two methods was basically equivalent.

### 3.3. CV

**Figure 11:**
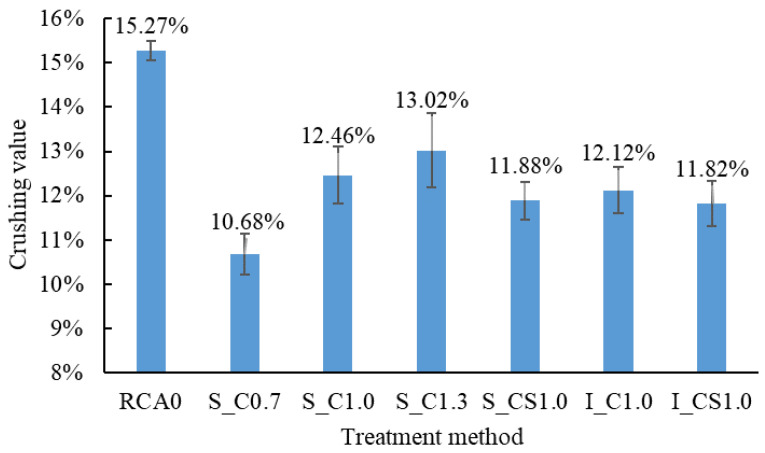
The CV of the RCA with different treatment methods.

In general, regardless of the treatment method and the type of slurry, the CV of all the treated RCA was all less than that of the raw RCA, as shown in Figure 11. The CV was decreased by up to 30.0% when the spraying method was used (S_C0.7). It meant that both the spraying treatment and the immersing treatment could enhance the RCA.

When pure cement slurry was used, all the CV of the treated RCA decreased. The enhancing effect of the pure cement slurry was also reported in [28,29], and the CV could be decreased by 14.6% (ordinary Portland cement (32.5) was used, and W/B was 1.0) [30], which was similar to our result. The CV decreased with the decrease in the W/B. The reason was that a hardened cement slurry coating was formed on the surface of the RCA and the strength of the coating was higher than the old mortar [33]; furthermore, more slurry was absorbed when the W/B was lower, as shown in Figure 9, which resulted in a thicker coating on the RCA. Therefore, the strength of the RCA was higher. By comparing S_CS1.0 with S_C1.0, when the SF was incorporated in the slurry, more slurry was absorbed, and the strength of the hardened coating was higher than that of pure cement slurry, which led to a higher strength of S_CS1.0.

By comparing S_C1.0 with I_C1.0 and S_CS1.0 with I_CS1.0, the difference in the CV between the spraying method and the immersing method was not significant. According to Figure 10, the enhancement of the RCA could be attributed to the coating and the strengthened old mortar. By using the two treatment methods, the coating and the strengthened old mortar were nearly the same, and so was the CV of the RCA. Based on the CV results, the spraying method was approximately equivalent to the immersing method, but it was more economical and efficient by using the spraying method.

### 3.4. WA

**Figure 12:**
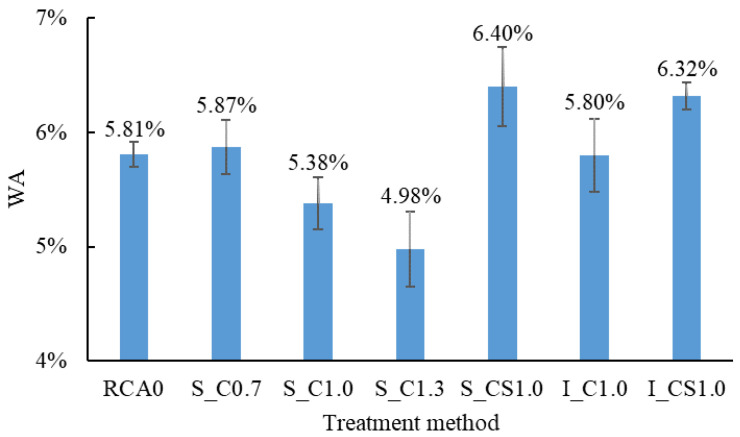
The results of the WA of the RCA.

Figure 12 shows the WA of the RCA treated by different methods. It was clearly represented that, with the increase in the W/B, the WA of the RCA treated by the spraying method decreased. When W/B was 0.7, the WA of S_C0.7 even slightly exceeded that of the raw RCA. When the SF was incorporated, the WA of the RCA was obviously increased for both the spraying method and the immersing method. The WA of S_CS1.0 and I_CS1.0 was nearly the same, and the WA of S_C1.0 was even lower than that of I_C1.0. It meant that, in terms of WA, the spraying method was equivalent or even superior to the immersing method. In order to lower the WA, the W/B should be not less than 1.0. According to the classification of the RCA, in terms of WA, the grade of the RCA was improved from III to II (less than 5%) when the spraying method was used, and the W/B was 1.3.

**Figure 13:**
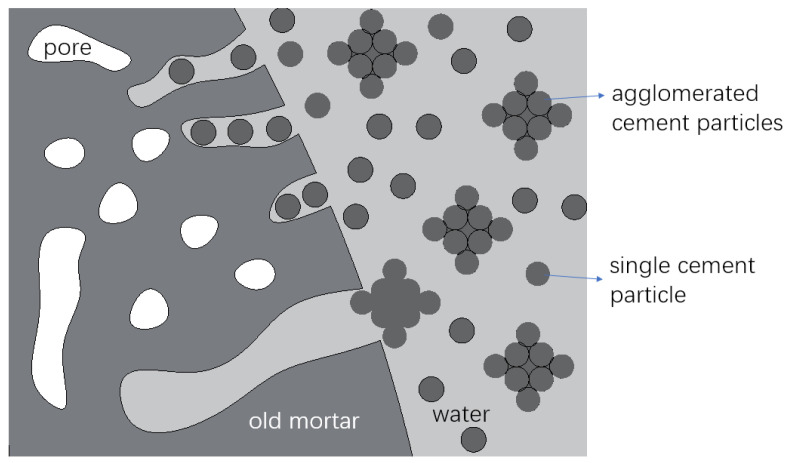
Schematic of the surface of the RCA treated by cement slurry.

Similar results were also reported in [30]. The mechanism was discussed as follows: When the higher W/B slurry was used, there were more free water and fewer agglomerated cement particles in the slurry. Hence, more water could permeate into the old mortar, and more cement particles transmitted into the pores of the old mortar with the water [38], as shown in Figure 13. Therefore, the thickness of the strengthened old mortar would be larger, which resulted in less water permeating into the old mortar of the RCA during the WA test. Moreover, the lower W/B and the incorporation of SF could lead to a larger thickness coating [27,36]; therefore, more water was absorbed [38,39].

### 3.5. DWA

**Figure 14:**
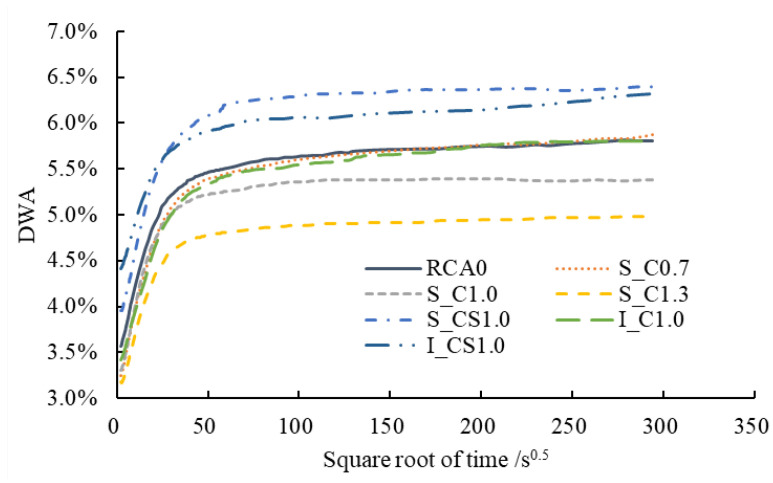
The DWA of the RCA during 24 h.

**Figure 15:**
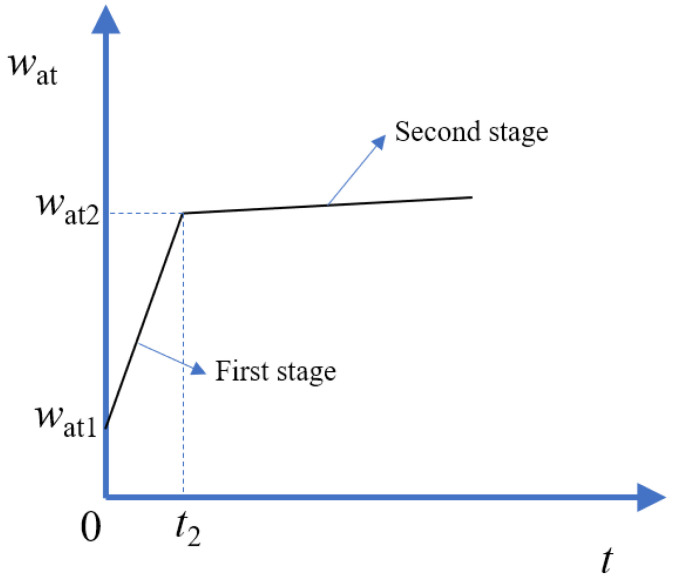
Schematic of the two-stage model of the RCA.

The DWA results are shown in Figure 14. The results of the first 5 s were not taken into account to avoid the influence of the artificial disturbance. According to Figure 14, the evolution of the DWA of the RCA contained two stages. At first, the water absorption increased dramatically; then, the water absorption tended to be steady. A two-stage model, which was usually adopted to analyze the evolution of water absorption of cement-based materials, could be used to depict the evolution of the DWA of the RCA [40]. During the first stage, the infiltration of water was mainly caused by capillary pores (the radius was less than 10 μm), and the water absorption rate was faster [41]. During the second stage, the infiltration of water was mainly caused by blowholes (the radius was larger than 10 μm), and the water absorption rate was also slowed down. Figure 15 shows the schematic of the two-stage model. Based on the two-stage model, two straight lines were used to fit the two stages, and the functions are listed in Table 5. The influence of the bubbles was eliminated by shaking the RCA at the beginning of the test, and the data were recorded after 5 s. The evolution of the DWA was not recorded during the first 5 s; therefore, at the very beginning of the first stage, the water absorption was not equal to 0. By extending the straight line of the first stage to the vertical axis, the water absorption at the very beginning of the test was determined, as shown in Table 5, and the saturation degree (SD_1_ = *w*_at1_/WA) was also calculated. It could be found that once the RCA was put into water, the saturation degree exceeded 50% immediately. The duration of the first stage, *t*_2_, was about 9–15 min. At the end of the first stage, the saturation degree (SD_2_ = *w*_at2_/WA) exceeded 90%. Similar results were also reported in [42,43]. It meant that the evolution of the DWA was not changed when the RCA was treated with cement-based slurry.

### 3.6. Discussion

**Figure 16:**
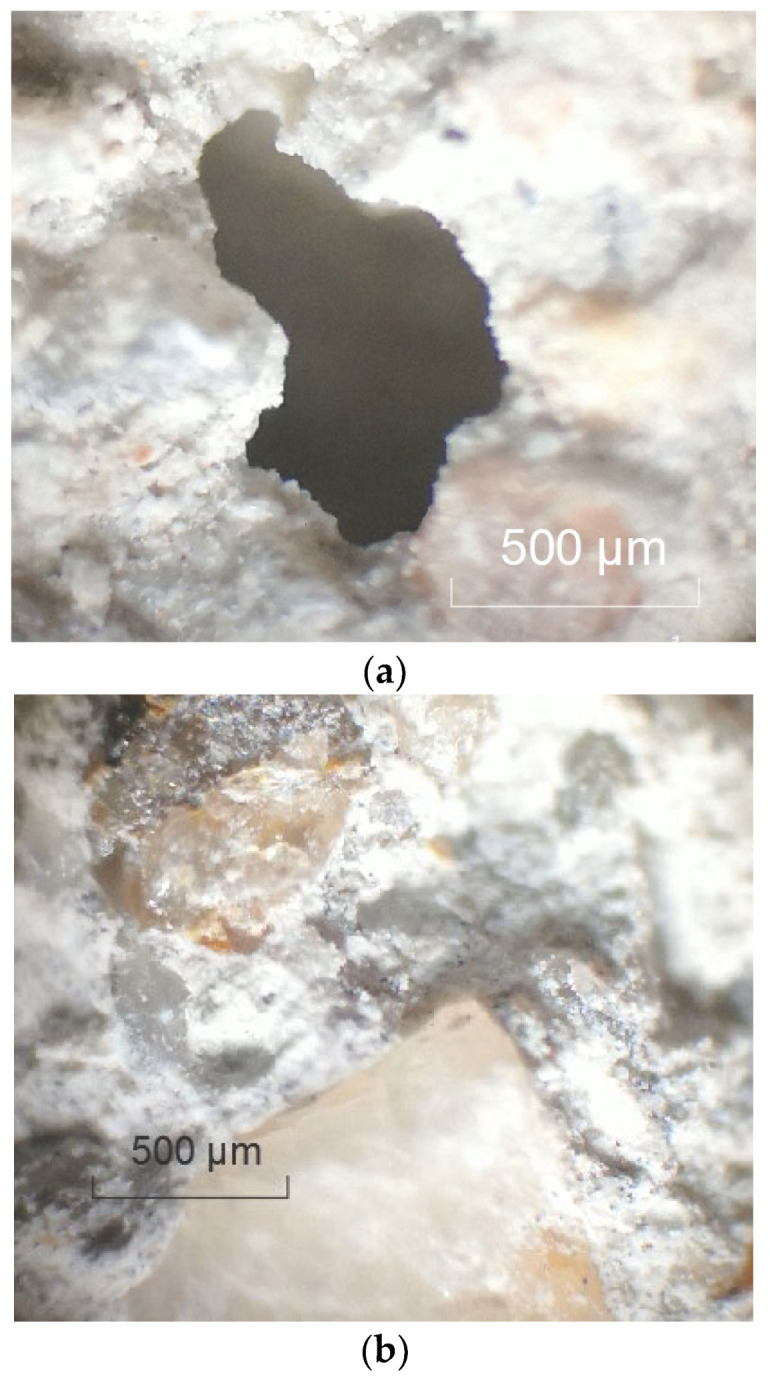
Images of the raw RCA obtained with optical microscope (magnification: 40). (**a**) Pore; (**b**) adhered mortar.

**Figure 17:**
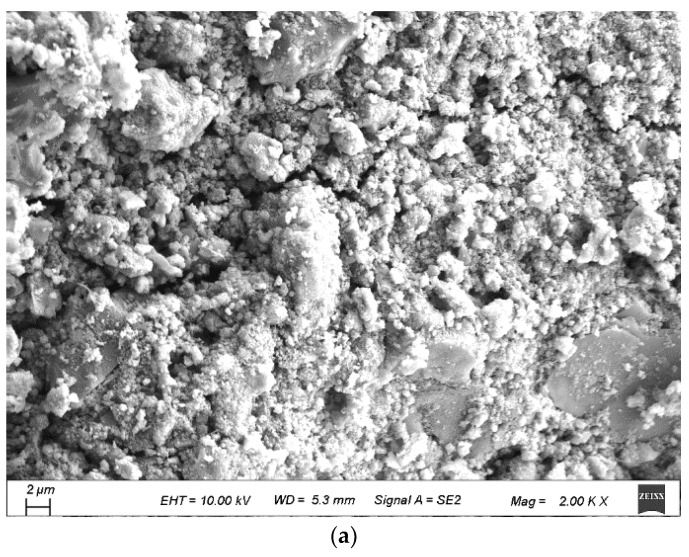

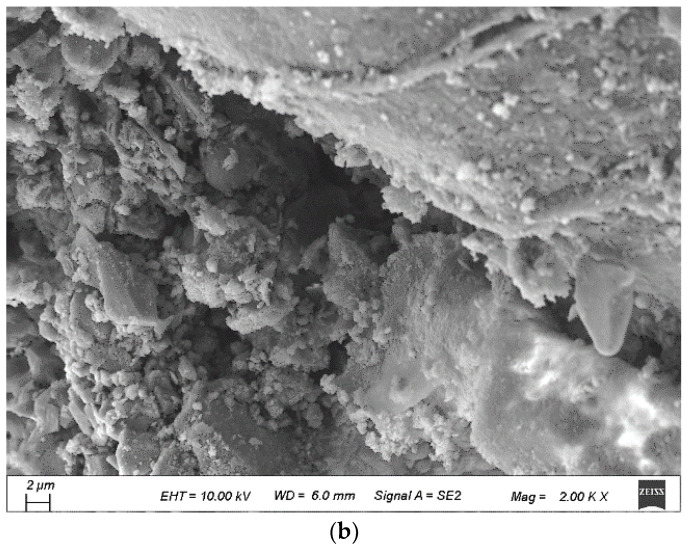
Images of the raw RCA obtained with SEM. (**a**) Micro-crack; (**b**) micro-pore.

In order to further understand the properties of the raw RCA and the influence of the spraying method, micro-morphology of the raw RCA was obtained, as shown in Figure 16 and Figure 17. The surface of the RCA was coarser than the natural aggregate due to the adhered mortar, and pores could be observed. It explained why the WA and the CV were higher.

When the cement-based slurry was used to strengthen the RCA, partial particles penetrated into the pores and cracks of the old mortar, and partial particles stuck on the surface of the RCA. The strengthening mechanisms were cement reaction and Pozzolanic reaction, as shown in Equations (6) and (7) [31].

(6) $$C_{3}S+H\to C-S-H+\mathrm{CH}$$

(7) CH+S→C−S−H

**Figure 18:**
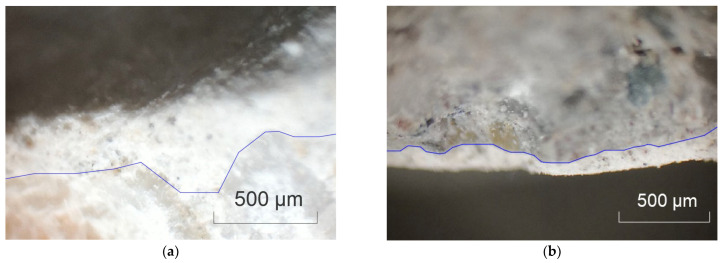

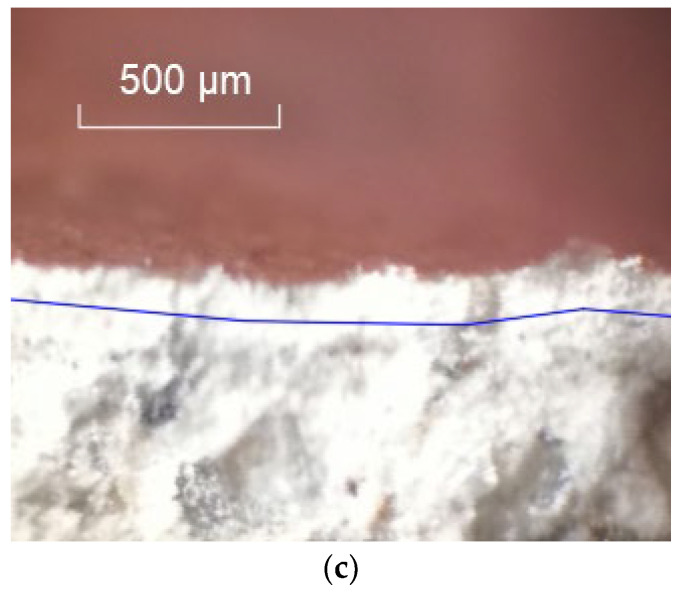
Cross-sections of the treated RCA (magnification: 40). (**a**) S_C0.7; (**b**) S_C1.0; (**c**) S_C1.3.

**Figure 19:**
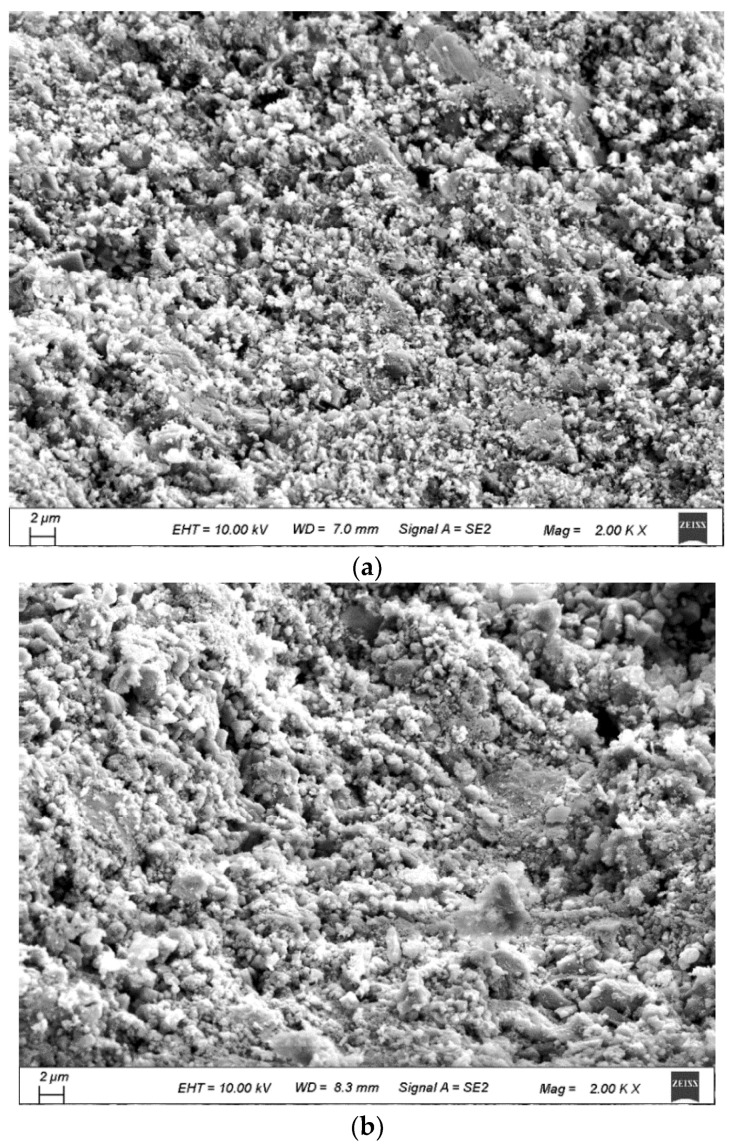

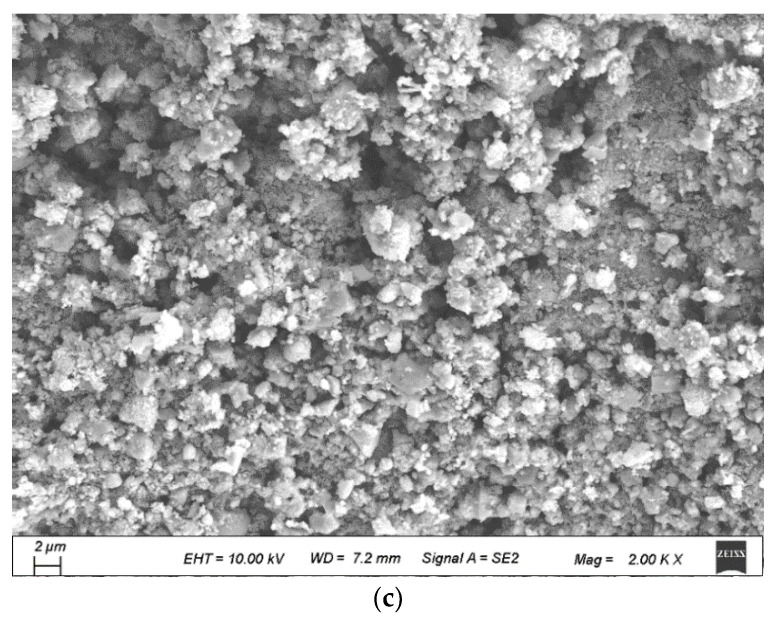
Surfaces of the treated RCA. (**a**) S_C1.0; (**b**) I_C1.0; (**c**) S_CS1.0.

Therefore, the strengthened old mortar layer and the cement-based coating were formed [44], and the RCA was strengthened, as shown in Figure 8, Figure 10 and Figure 13. The micro-morphologies of the RCA are shown in Figure 18, and a 0.2 mm thick coating was reported in [27]. The thickness of the coating was related to the W/B, and the smaller the W/B was, the larger the thickness was. Furthermore, the cement-based slurry tended to concentrate in depressions which caused the surface of the RCA to become smoother. According to Figure 19, more C − S − H was found on the surface of the treated RCA, and the surfaces were densified; hence, the strength of the RCA was improved. Although the strength of the RCA could be improved, the coating was porous (as shown in Figure 19) and hydrophilic (as proved in the WA test), which could not prevent the penetration of water; therefore, the WA of the RCA could not be decreased, and a similar result could be found in [28].

According to the results of the DWA of the RCA, the water absorption rate of the RCA was very fast, and after about 30 s, the saturation degree exceeded 60%, while Yang yunqi [42] reported that the saturation reached more than 67.7% within 1 min. On the other hand, the penetration of cement particles depended on the penetration of water. Accompanying the rapid penetration of water, cement particles filled the pores and cracks of the old mortar of the RCA. When the pores and cracks had been filled with cement particles, other particles could not penetrate into the old mortar; therefore, extending treatment time could not increase the thickness of the strengthened mortar layer, and the strengthening layer of the old mortar was limited [33]. Furthermore, extending treatment time also could not influence the thickness of the coating. Therefore, the influences of the spraying method and the immersing method were basically equivalent.

## 4. Conclusions

- A batch spraying method, using cement-based slurry, was proposed to treat the RCA. Based on the slurry absorption, when the flow rate was 5 g/(s∙kg), the RCA was basically saturated within three spraying cycles, and the slurry and time required were about 5% of the mass of the RCA and 1 min, respectively;
- The strength of the RCA was improved regardless of the treatment method and the W/B of the slurry. The CV decreased by up to 30% when the spraying method was used with a W/B of 0.7. When the SF was incorporated, the crushing value of the RCA could be further decreased;
- The WA of the RCA decreased with the increase in the W/B. To decrease the WA of the RCA, the W/B should be no less than 1.0. The WA decreased by up to 14.3% when the spraying method was used with a W/B of 1.3. With the incorporation of the SF, the WA of the RCA showed an obvious increase regardless of the treatment method;
- The DWA test showed that the water absorption rate was very rapid, and the evolution of the water absorption was not affected by the spraying treatment or the immersing treatment using cement-based slurry. When the RCA entered the water, the saturation degree reached more than 50%. After about 9–15 min, the saturation degree of the RCA reached more than 90%;
- In terms of the CV, WA and AD, the effects of the spraying method and the immersing method were basically equivalent. When the spraying method was used, only about 1 min and a small amount of slurry (about 5% of the RCA mass) were required to treat the RCA. It was recommended that pure cement slurry should be used and the W/B should be 1.3 when the spraying method was adopted to treat the RCA, and the comprehensive grade could be enhanced from III to II.

## Figures and Tables

**Table 1:** Properties of the raw RCA.

Property	Apparent Density (kg/m^3^)	Water Absorption (%)	Crushing Value (%)	Soundness (%)	Particle Size (mm)
value	2687.3	5.81	15.27	4.9	9.5~19
grade	I	III	II	I	-

**Table 2:** The main chemical compositions of cement.

Composition	CaO	SiO_2_	Al_2_O_3_	Fe_2_O_3_	MgO	SO_3_
Percentage (wt%)	59.26	20.47	6.31	4.08	2.01	2.23

**Table 3:** Chemical compositions of the silica fume.

Composition	SiO_2_	Al_2_O_3_	Fe_2_O_3_	MgO	CaO	Na_2_O
Percentage	95%	1.0%	0.9%	0.7%	0.3	1.3

**Table 4:** Proportions of cement-based slurries and treatment methods.

Number	Water to Binder Ratio (W/B)	Binder		Treatment Method	Abbreviation
		Cement Ratio (wt%)	Silica Fume Ratio (wt%)		
1	0.7:1	100	0	spraying	S_C0.7
2	1:1	100	0	spraying	S_C1.0
3	1.3:1	100	0	spraying	S_C1.3
4	1:1	92	8	spraying	S_CS1.0
5	1:1	100	0	immersing	I_C1.0
6	1:1	92	8	immersing	I_CS1.0

**Table 5:** Functions and key parameters of the two-stage model of the RCA.

RCA	First Stage	Second Stage	*t*_2_ s	*w_at_*_2_	SD_1_	SD_2_
RCA0	*w_at_* = 0.0009 $\sqrt{t}$ + 0.0336 R^2^ = 0.995	*w_at_* = 9 × 10^−6^ $\sqrt{t}$ + 0.0557 R^2^ = 0.9598	615	5.59%	57.8%	96.3%
S_C0.7	*w_at_* = 0.001 $\sqrt{t}$ + 0.0301 R^2^ = 0.996	*w_at_* = 1 × 10^−5^ $\sqrt{t}$ + 0.0549 R^2^ = 0.9831	628	5.52%	51.3%	94.0%
S_C1.0	*w_at_* = 0.0009 $\sqrt{t}$ + 0.0306 R^2^ = 0.9972	*w_at_* = 2 × 10^−5^ $\sqrt{t}$ + 0.0514 R^2^ = 0.8715	559	5.19%	56.9%	96.4%
S_C1.3	*w_at_* = 0.0007 $\sqrt{t}$ + 0.0295 R^2^ = 0.9941	*w_at_* = 5 × 10^−6^ $\sqrt{t}$ + 0.0484 R^2^ = 0.9706	740	4.85%	59.2%	97.5%
S_CS1.0	*w_at_* = 0.0009 $\sqrt{t}$ + 0.037 R^2^ = 0.9933	*w_at_* = 4 × 10^−6^ $\sqrt{t}$ + 0.0628 R^2^ = 0.835	829	6.29%	57.8%	98.3%
I_C1.0	*w_at_* = 0.0007 $\sqrt{t}$ + 0.0324 R^2^ = 0.9968	*w_at_* = 2 × 10^−5^ $\sqrt{t}$ + 0.0529 R^2^ = 0.9757	909	5.35%	55.9%	92.2%
I_CS1.0	*w_at_* = 0.0007 $\sqrt{t}$ + 0.0424 R^2^ = 0.9966	*w_at_* = 1 × 10^−5^ $\sqrt{t}$ + 0.0589 R^2^ = 0.9639	572	5.91%	67.1%	93.6%

## Data Availability

Data are available on request from the authors.
